# Inhibition of HDAC1 alleviates monocrotaline-induced pulmonary arterial remodeling through up-regulation of miR-34a

**DOI:** 10.1186/s12931-021-01832-7

**Published:** 2021-08-31

**Authors:** Fangwei Li, Dan Wang, Hong Wang, Lijun Chen, Xilu Sun, Yixin Wan

**Affiliations:** Department of Respiratory Medicine, Lanzhou University Second Hospital, No. 82, Cuiying Men, Lanzhou, 730030 Gansu People’s Republic of China

**Keywords:** HDAC1, Extracellular matrix, miR-34a, Pulmonary arterial hypertension

## Abstract

**Background:**

It has been found that up-regulation of histone deacetylases 1 (HDAC1) is involved in the development of pulmonary arterial hypertension (PAH). However, it is still unclear whether inhibition of HDAC1 suppresses the development of PAH via restoring miR-34a level in monocrotaline (MCT)-induced PAH rats.

**Methods:**

PAH rat models were induced by intraperitoneal injection of MCT. HDAC1 was suppressed by intraperitoneal injection of the class I HDAC inhibitor MS-275, and miR-34a was over-expressed via tail vein injection of miR-34a agomiR.

**Results:**

HDAC1 protein was significantly increased in MCT-induced PAH rats; this was accompanied with down-regulation of miR-34a and subsequent up-regulation of matrix metalloproteinase 9 (MMP-9)/tissue inhibitor of metalloproteinase 1 (TIMP-1) and MMP-2/TIMP-2. Administration of PAH rats with MS-275 or miR-34a agomiR dramatically abolished MCT-induced reduction of miR-34a and subsequent up-regulation of MMP-9/TIMP-1 and MMP-2/TIMP-2, finally reduced extracellular matrix (ECM) accumulation, pulmonary arterial remodeling, right ventricular systolic pressure (RVSP) and right ventricle hypertrophy index (RVHI) in PAH rats.

**Conclusions:**

HDAC1 contributes to the development of MCT-induced rat PAH by suppressing miR-34a level and subsequently up-regulating the ratio of MMP-9/TIMP-1 and MMP-2/TIMP-2. Inhibition of HDAC1 alleviates pulmonary arterial remodeling and PAH through up-regulation of miR-34a level and subsequent reduction of MMP-9/TIMP-1 and MMP-2/TIMP-2, suggesting that inhibition of HDAC1 might have potential value in the management of PAH.

## Background

Pulmonary arterial hypertension (PAH) is defined as a hemodynamic state of the resting mean pulmonary arterial pressure equal to or more than 25 mmHg, leading to right heart failure and ultimately death [1]. Pulmonary vascular remodeling is the prominent hallmark of PAH, in which the accumulation of extracellular matrix (ECM) with increased collagen deposition is an indispensable change across the vascular wall [2, 3]. Excessive accumulation of ECM is convinced to occur due to the deregulation of multiple signaling pathways in pulmonary arterial smooth muscle cells (PASMCs) [4]. Therefore, it is important to inhibit the cascades responsible for collagen synthesis or activate the signaling pathways associated with collagen degradation in the prevention and treatment of PAH.

Histone acetylation is dynamically regulated by histone acetyltransferases (HATs) and histone deacetylases (HDACs), playing an important role in controlling gene expression [5]. HDACs remove acetyl groups from the lysine residues of histones and cause dense crispation of chromatin, leading to the suppression of gene transcription; while HATs result in the opposite process [6]. There are 18 HDACs which are grouped into four classes and HDAC1 belongs to the class I HDACs [6]. Class I HDACs (HDAC1, 2, 3, and 8) play an important role in controlling cell cycle regulation, ECM accumulation and tissue development by deacetylating substrates and then regulating gene expression [7–9]. Studies have shown that the overall activity of HDACs is determined by the expression level of HDAC1 to some extent [10].

Recent studies have found that HDAC1 is up-regulated in lung tissues of patients with PAH and model rats [11, 12]; selective class I HDAC inhibitors reverse monocrotaline (MCT) and hypoxia-induced pulmonary arterial remodeling and PAH by reducing HDAC1 expression [13]. Class I HDACs participate in ECM remodeling of various tissues through multiple molecular mechanisms [9, 14]. In addition, studies have suggested that HDAC1 inhibits miR-34a expression directly or indirectly [15, 16] and down-regulation of miR-34a plays an important role in the pathogenesis of PAH [17, 18]. However, whether inhibition of HDAC1 alleviates pulmonary arterial remodeling and PAH via suppressing excessive ECM accumulation is not defined. In addition, whether inhibition of HDAC1 restores miR-34a expression and thereby reduces excessive ECM accumulation needs further exploration. In the current study, rat models of MCT-induced PAH were used to evaluate the effects of HDAC1 on ECM remodeling of the pulmonary artery and to further explore its potential molecular mechanisms.

## Methods

### Animals

Forty male Sprague–Dawley (SD) rats weighing 150–200 g used in this study were purchased from Lanzhou Veterinary Research Institute, Chinese Academy of Agricultural Sciences. Rats were kept under standard conditions (18–22 °C and 40–60% humidity) in a specific pathogen free animal laboratory, and they had free access to food and water according to the U.S. National Research Council’s Guide for the Care and Use of Laboratory Animals. Forty rats were divided into four groups randomly (n = 10 in each group): control group, MCT group, MCT + MS-275 group, and MCT + miR-34a agomiR group.

### Chemical reagents

MCT (Sigma-Aldrich, St. Louis, MO, USA) was dissolved in 1 mol/L HCL and then titrated with 1 mol/L NaOH to pH 7.4 with a final concentration of 30 mg/mL. Class I HDAC inhibitor MS-275 (Selleck Chemicals, USA) was dissolved in dimethyl sulfoxide (DMSO) and then diluted with normal saline to a final concentration of 6 mg/mL. miR-34a agomiR (GenePharm, Shanghai, China) was diluted with diethyl pyrocarbonate (DEPC) to a final concentration of 25 μM.

### Generation of rat PAH and reagent intervention models

The rat PAH model was induced by intraperitoneally injection of MCT (60 mg/kg) on day 1. MS-275 (3 mg/kg) was administrated to rats by intraperitoneally injection every other day for 28 days after MCT injection. miR-34a agomiR (25 μM) was given to rats via tail vein injection on days 1, 7, 14, 21, and 28 respectively after MCT injection.

### Hemodynamic and right ventricular hypertrophy evaluation

After 28 days of MCT injection, all survived rats were anesthetized with 10% chloral hydrate (3–5 mL/kg) via intraperitoneal injection. Then the rats were put in supine position and a polyvinyl catheter filled with heparin saline solution were inserted into the right ventricle via the isolated right external jugular vein. Finally, the right ventricle systolic pressure (RVSP) was measured using a Grass polygraph (Power Lab, Australia). After completion of hemodynamic measurements, the hearts and lungs were harvested. Then the right ventricle (RV) was dissected from the left ventricle (LV) plus interventricular septum (S). Each part was weighed separately to calculate the right ventricular hypertrophy index (RVHI), which is the ratio of the weight of RV to the LV plus S [RV/(LV + S)].

### Histological analysis

The harvested right upper pulmonary lobes were fixed using 4% paraformaldehyde for 24 h at room temperature and then embedded in paraffin wax. The paraffin embedded tissue blocks were sectioned at 5 μm thickness and then stained with hematoxylin and eosin (HE) staining as well as Masson’s trichrome staining for morphological analysis. To evaluate the degree of pulmonary arterial remodeling, the distance between the inner and outer elastic fiber layers (medial wall thickness, MT) and the average diameter of the outer elastic fiber layer (external diameter, ED) were measured in HE staining under a light microscope (×400 magnification). Then the percentage of medial wall thickness (%MT) of vessels was calculated as follows [19, 20]: %MT = (2 × MT/ED) × 100%.

### Immunofluorescence staining

The paraffin sections were prepared using the same method as in HE staining, on which the immunofluorescence staining of rat lung tissues was performed. The sections were blocked with 3% bovine serum albumin (BSA) to avoid nonspecific immunoreactions and then incubated overnight at 4 °C with primary antibody of HDAC1 (CST, Beverly, MA, USA; 1:1000). After being washed three times, the sections were incubated at 37 °C for 2 h with goat anti-rabbit secondary antibody (Sigma-Aldrich; 1:5000). Subsequently, the sections were stained by 4′,6-diamidino-2-phenylindole (DAPI) for 5 min at room temperature. In addition, negative control immunofluorescence was conducted to show the auto-fluorescence using equal amount of phosphate buffered solution (PBS) instead of the primary antibody and secondary antibody. Finally, fluorescence images were randomly scanned using a confocal laser scanning microscope at ×400 magnification.

### Cell culture

Primary cultured PASMCs were obtained from the pulmonary arteries of 4- to 5-week-old male SD rats. All animal experiments were approved by the Laboratory Animal Care Committee of Lanzhou University Second Hospital and performed in accordance with the Guidelines for Animal Experimentation of Lanzhou University Second Hospital. The pulmonary arteries were rapidly removed from euthanized rats and the layers of adventitia as well as endothelium were removed gently. Then the remaining smooth muscle layer was minced (~ 1 mm^2^) and placed into a culture flask with Dulbecco's Modified Eagle Medium (DMEM, Gibco, Grand Isle, NY, USA) containing 10% fetal bovine serum (FBS, Sijiqing, HangZhou, China) as well as 1% antibiotics (100 U/mL penicillin and 100 μg/mL streptomycin). The tissue blocks were cultured at 37 °C in an atmosphere of 95% air and 5% CO_2_. PASMCs were sub-cultured using 0.25% trypsin (Invitrogen, Carlsbad, CA, USA) when reaching 80% confluence, and cells between passages 4 and 6 were used for cell studies.

### siRNA transfection

Cell transfection was performed for knockdown the expression of HDAC1 using Lipofectamine™ 2000 transfection reagent (Invitrogen) according to the manufacturer's protocols. PASMCs were cultured in a 6-well plate until reaching 30–40% confluence. The nucleotides of HDAC1 siRNA were used at a final concentration of 100 nM in DMEM (without serum or antibiotics) for 6 h and complete DMEM with 10% FBS (without antibiotics) for 48 h in a 37 °C, 5% CO_2_ humidified incubator. Transfection efficiency of siRNA was determined using immunoblotting.

### ELISA

The collagen I level in harvested peripheral lung tissues was measured using a rat collagen I enzyme-linked immunosorbent assay (ELISA) kit (Xitang, Shanghai, China) according to the manufacturer’s instructions. Briefly, the lung tissues were homogenized in 0.9% NaCl on ice and centrifuged at 6000 rpm for 20 min at 4 °C. Then the supernatant was collected and the concentration of sample was calculated automatically by the instrument software according to the measured optical density values. Duplicate samples were assayed, and all results were reported as the mean values.

### Immunoblotting

The left lung tissues were homogenized in RIPA lysis buffer (HEART, Xi'an, China) containing protease inhibitors, phosphatase inhibitors as well as phenylmethylsulfonyl fluoride (PMSF), and centrifuged at 12,000 rpm at 4 °C for 20 min. Then the supernatant was collected as total protein. The amount of protein in each extract was measured using BCA protein assay kit (Pierce Biotechnology, Rockford, IL, USA). Subsequently, equal amounts of protein extracts were separated on SDS-PAGE gel (10%) and transferred to polyvinylidene fluoride (PVDF, Bio-Rad, Richmond, CA, USA) membrane using wet transfer. After washing, the blots were incubated at 4 °C overnight with the primary antibodies against HDAC1 (CST; 1:1000), HDAC2 (CST; 1:1000), HDAC3 (CST; 1:1000), tissue inhibitor of metalloproteinase 1 (TIMP-1, Abcam, Cambridge, UK; 1:1000), TIMP-2 (Abcam; 1:1000) and β-actin (Santa Cruz, Dallas, TEX, USA; 1:500). Then the horseradish peroxidase-conjugated goat anti-rabbit IgG (Sigma-Aldrich; 1:5000) was used as the secondary antibodies. Finally, immunoreactive bands were visualized by Super Signal West Pico Chemiluminescent Substrate (Pierce Biotechnology) and quantified by Quality One software (Bio-Rad).

### qRT-PCR

Total RNA was extracted from lung tissues using TRIzol reagent (Invitrogen) according to the manufacturer’s instructions. U6 small nuclear RNA was chosen for endogenous control of miR-34a. Complementary DNA (cDNA) was synthesized using the RevertAid First Strand cDNA Synthesis Kit (Thermo, Waltham, MA, USA) and quantitative real-time polymerase chain reaction (qRT-PCR) was carried out using the Maxima SYBR Green/ROX qPCR Master Mix (Thermo). miR-34a was quantified by measuring cycle threshold (Ct) values and normalized using 2^−ΔΔCt^ method relative to U6. Primers (Sangon Biotech, Shanghai, China) for miR-34a and U6 were as follows:

miR-34a RT, 5′-GTCGTATCCAGTGCGTGTCGTGGAGTCGGCAATTGCACT

GGATACGACTAGGGC-3′;

miR-34a forward, 5′-CGCGAATCAGCAAGTATAACTGCC-3′;

miR-34a reverse, 5′-CGTATCCAGTGCGTGTCGTGG-3′;

U6 RT, CGCACTGGATACGACGGCATTCT;

U6 forward, CTCGCTTCGGCA GCACA;

U6 reverse, AACGCTTCACGAATTTGCGT.

### HDAC activity

Nuclear extracts were obtained from rat lung tissues (20 mg) using the Nuclear Extract kit (Active Motif, Carlsbad, CA, USA) according to the manufacturer's instructions. HDAC activity was measured in the nuclear extracts with the Fluorometric HDAC Activity Assay Kit (Abnova, walnut, Los Angeles, USA) in accordance with the manufacturer’s instructions. Briefly, 15 μg nuclear extracts was diluted in assay buffer and trichostatin A, a classic HDAC inhibitor as the negative control. Then the HDAC reaction was initiated by addition of fluorometric substrate and the mixture was incubated at 37 °C for 30 min. Finally, the reaction was stopped by adding lysine developer for 30 min and the sample was measured using a fluorescence plate reader with excitation at 380 nm and emission at 460 nm (Bio-Rad). The HDAC activity was expressed as relative fluorescence units.

### Gelatin zymography

The enzymatic activity of matrix metalloproteinase 2/9 (MMP-2/9) was determined by gelatin zymography. Rat lung tissues were homogenized in Tris–HCl buffer containing 50 mM Tris–HCl pH 7.6, 150 mM NaCl, 5 mM CaCl_2_, and 0.1% Triton X-100. Equal amounts of protein were separated on gelatin zymography gel by electrophoresis. Next, the gel was incubated for 48 h in development buffer containing 50 mM Tris–HCl pH 7.6, 5 mM CaCl_2_, and 1 μM ZnCl_2_ at 37 °C. Then the gel was stained with 0.5% Coomassie Blue R-250 for 3 h and destained in destaining buffer containing 30% MeOH and 10% acetic acid. Finally, Gel Doc™ XR (Bio-Rad) was used to visualize and analyze the resulting bands.

### Statistics

The statistical analysis was performed by SPSS 13.0 software (SPSS Inc., Chicago, IL, USA). Values were presented as mean ± standard deviation. Group comparisons were performed using one-way analysis of variance (ANOVA) followed by Dunnett post hoc test (between the control group and treatment groups) or Student–Newman–Keuls post hoc test (among different groups). *P* < 0.05 was considered to be statistically significant.

## Results

### Inhibition of HDAC1 ameliorates the increase of RVSP and RVHI in MCT-induced PAH rats

**Fig. 1:**
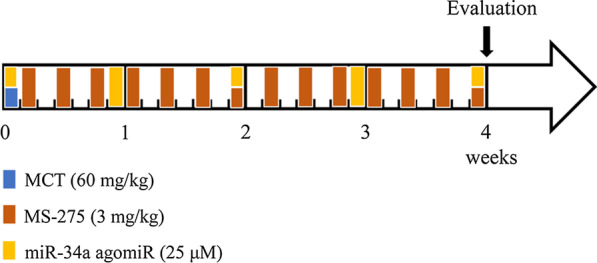
Protocol for generation of rat pulmonary arterial hypertension (PAH) and reagent intervention models. The rat PAH model was induced by intraperitoneally injection of monocrotaline (MCT) (60 mg/kg) on day 1. MS-275 (3 mg/kg) was administrated to rats by intraperitoneally injection every other day for 28 days after MCT injection. miR-34a agomiR (25 μM) was given to rats via tail vein injection on days 1, 7, 14, 21 and 28 respectively after MCT injection

**Fig. 2:**
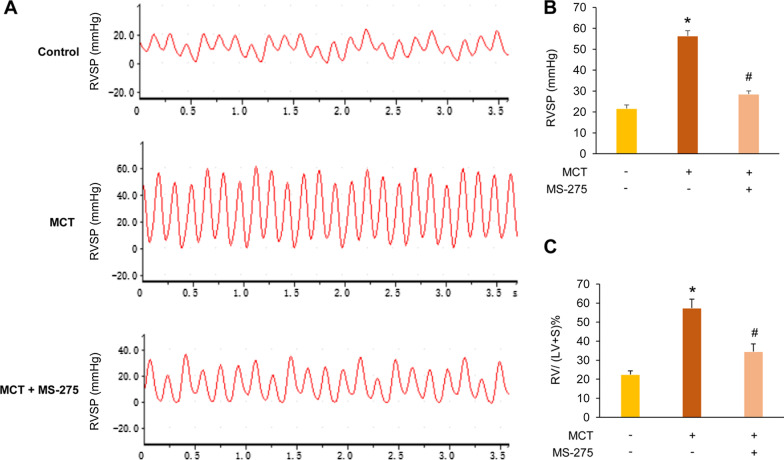
MS-275 alleviates monocrotaline (MCT)-induced pulmonary arterial hypertension (PAH) in rats. **a** Right ventricular systolic pressure (RVSP) waveform. **b** Changes of RVSP in each group (n = 6–8). **c** Changes of right ventricular hypertrophy index (RVHI) in each group, RVHI = RV/(LV + S) (n = 6–8). *P < 0.05 versus control group; ^#^*P* < 0.05 versus MCT group

Protocol for generation of rat PAH and reagent intervention models was shown in Fig. 1. After 28 days of MCT injection, the RVSP in MCT-treated rats was significantly increased compared with the control group (56.45 ± 2.54 mmHg versus 21.70 ± 1.70 mmHg, *P* < 0.05), suggesting that PAH was successfully induced by MCT in rats. However, treatment with class I HDAC inhibitor MS-275 after MCT injection dramatically reduced RVSP to 28.69 ± 1.42 mmHg (*P* < 0.05 versus MCT group), as depicted in Fig. 2A and B. Similar changes were observed in RVHI. Figure 2C showed that the RV/(LV + S) ratio was elevated from 0.23 ± 0.02 in the control to 0.57 ± 0.05 in MCT-induced PAH rats (*P* < 0.05). However, after administration of MS-275 in rats exposed to MCT, the RV/(LV + S) ratio was declined to 0.35 ± 0.04 (*P* < 0.05 versus MCT group). These results suggest that inhibition of HDAC1 suppresses the development of PAH.

### HDAC1 is increased in MCT-induced PAH rats

**Fig. 3:**
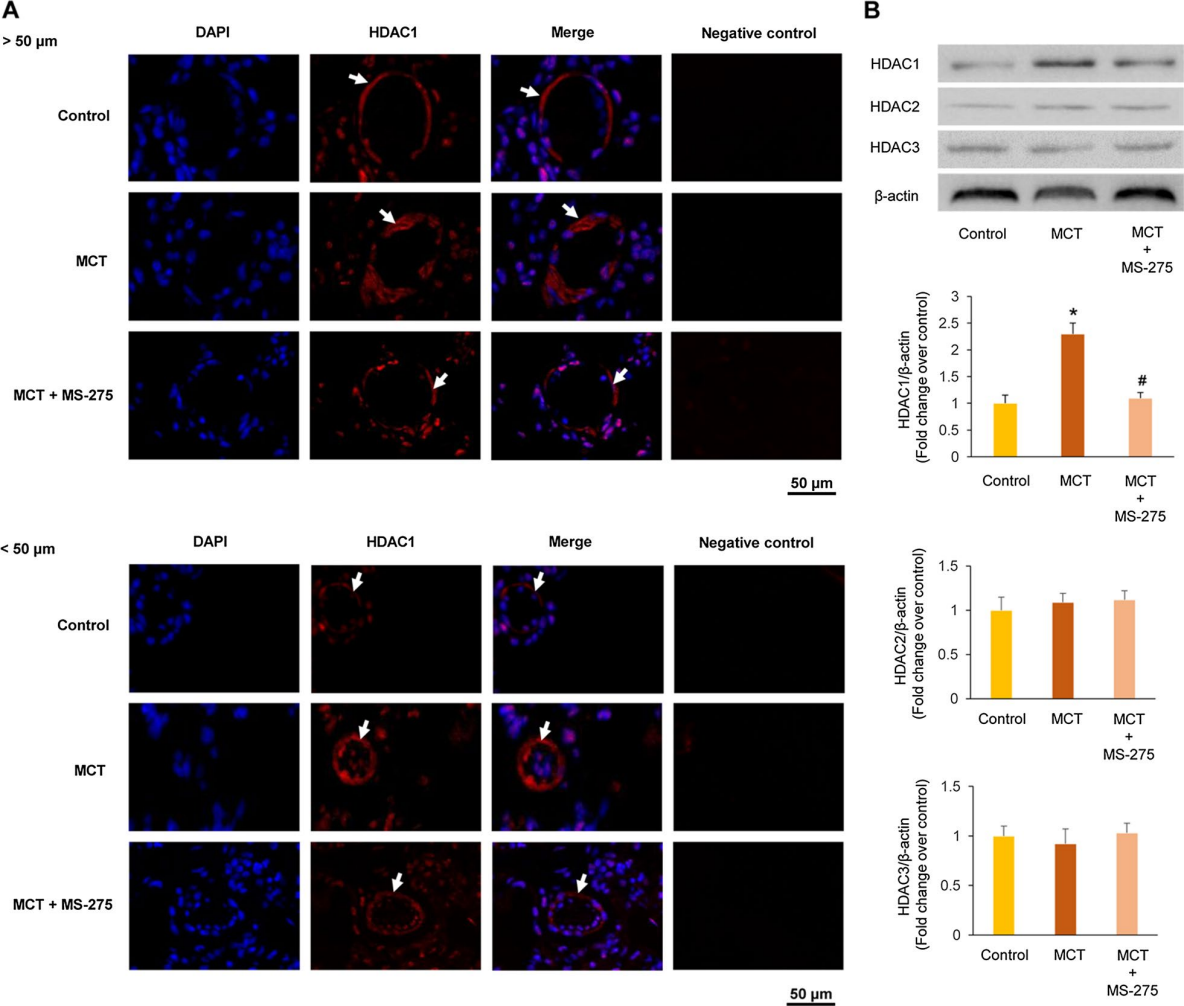
Histone deacetylases 1 (HDAC1) is up-regulated in monocrotaline (MCT)-induced pulmonary arterial hypertension (PAH) rats. **a** HDAC1 in the lung tissues from different groups was detected using immunofluorescence staining. **b** Protein levels of HDAC1/2/3 in the lung tissues from different groups were determined using immunoblotting (n = 5). *P < 0.05 versus control group; ^#^*P* < 0.05 versus MCT group

The protein level of HDAC1 in the lung tissues of MCT-treated rats was detected by immunofluorescence staining and immunoblotting to determine the potential role of HDAC1 in PAH. As indicated in Fig. 3A, the fluorescence-labeled HDAC1 in pulmonary vessels with diameters both greater than 50 µm and less than 50 µm of MCT-treated rats showed a significant increase compared with the control rats, while MS-275 treatment reduced fluorescence-labeled HDAC1 in pulmonary vessels with diameters both greater than 50 µm and less than 50 µm of MCT-treated rats. As expected in Fig. 3B, the protein level of HDAC1 was up-regulated to 2.30-fold in MCT-treated rats compared with the control rats (*P* < 0.05), while administration of MS-275 down-regulated HDAC1 level of MCT-treated rats to 1.15-fold over control (*P* < 0.05 versus MCT group). The protein levels of HDAC2 and HDAC3 remained unchanged in MCT-treated rats compared with the control rats (*P* > 0.05) and were not affected by administration of MS-275 in rats exposed to MCT (*P* > 0.05 versus MCT group). These results suggest that HDAC1 is significantly increased and can be inhibited by MS-275 in MCT-induced PAH rats.

### Inhibition of HDAC1 restores miR-34a expression in MCT-induced PAH rats and PASMCs

**Fig. 4:**
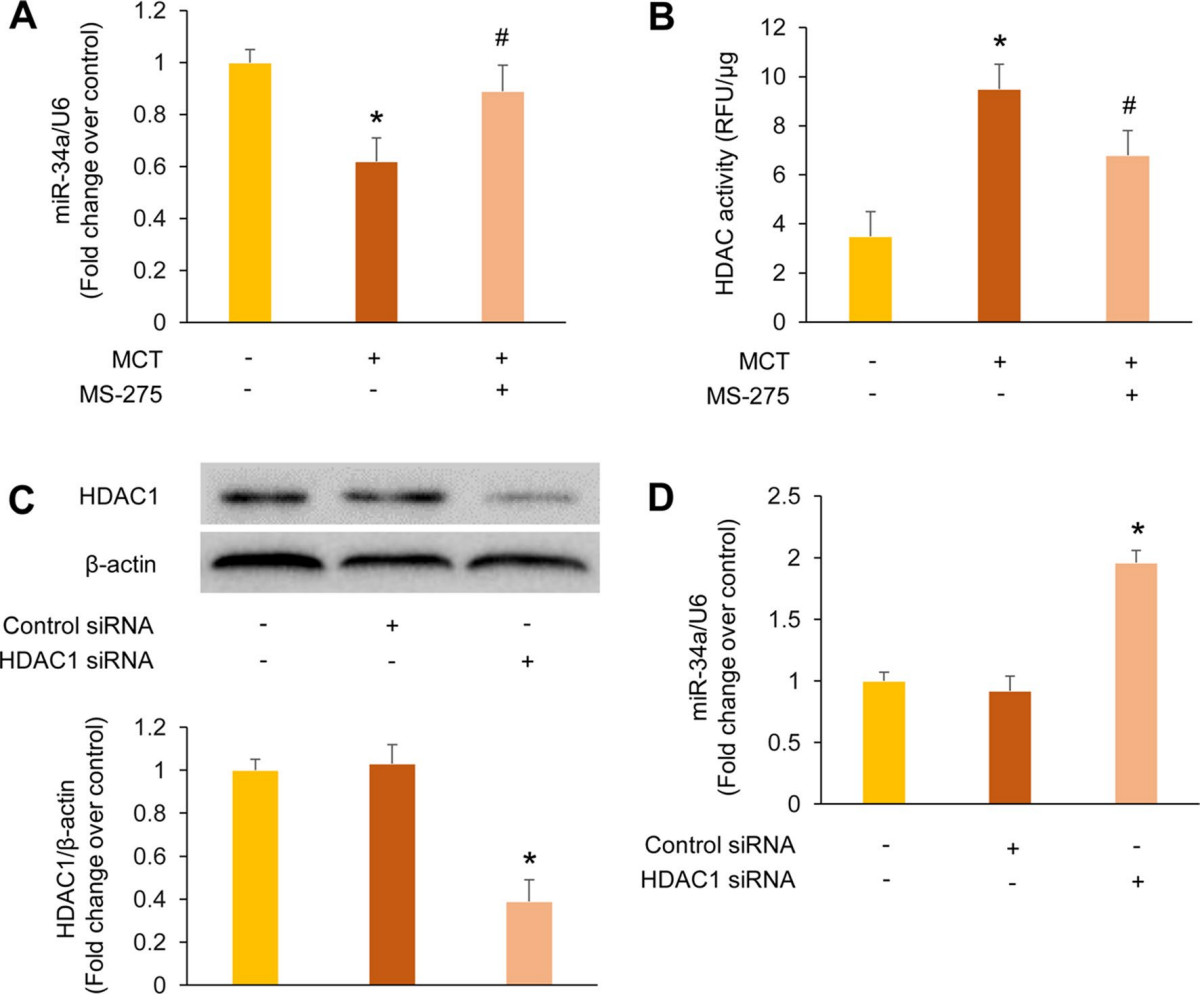
Inhibition of histone deacetylases 1 (HDAC1) restores miR-34a level in monocrotaline (MCT)-induced pulmonary arterial hypertension (PAH) rats and pulmonary arterial smooth muscle cells (PASMCs). **a** miR-34a level in lung tissues from different groups was determined using quantitative real-time polymerase chain reaction (qRT-PCR) (n = 3). **b** HDAC activity in lung tissues from different groups was measured with a fluorometric HDAC activity assay kit (n = 3). **c** Protein level of HDAC1 in PASMCs transfected with HDAC1-specific siRNA or control siRNA was examined using immunoblotting (n = 3). **d** miR-34a level in PASMCs transfected with HDAC1-specific siRNA or control siRNA was tested using qRT-PCR (n = 3). *P < 0.05 versus control group; ^#^*P* < 0.05 versus MCT group

To determine whether HDAC1 down-regulates miR-34a through deacetylation in MCT-induced PAH rats, the expression of miR-34a and the activity of HDAC were examined. As shown in Fig. 4A, MCT obviously reduced miR-34a level to 0.62-fold compared with control group (*P* < 0.05), while treatment of MCT-induced PAH rats with class I HDAC inhibitor MS-275 restored the miR-34a expression to 0.89-fold over control (*P* < 0.05 versus MCT group). Furthermore, the MCT-treated rats showed increased HDAC activity compared with the control group (9.52 ± 0.35 RFU/μg versus 3.62 ± 0.25 RFU/μg, *P* < 0.05), while administration of MCT-induced PAH rats with MS-275 significantly reduced HDAC activity to 6.8 ± 0.15 RFU/μg (*P* < 0.05 versus MCT group), as presented in Fig. 4B. These findings indicate that inhibition of HDAC1 restores miR-34a expression through reducing deacetylation in MCT-induced PAH rats.

In addition, HDAC1 siRNA was transfected into the rat primary cultured PASMCs to verify the specific role of HDAC1 in regulation of miR-34a. Figure 4C showed that HDAC1 siRNA reduced HDAC1 protein level to 39% of the control group (*P* < 0.05), whereas control siRNA did not affect HDAC1 protein level. Figure 4D presented that silencing of HDAC1 dramatically up-regulated miR-34a level, which increased to 1.96-fold compared with control group (*P* < 0.05), while the non-targeting siRNA had no effect on miR-34a expression. These results suggest that knockdown of HDAC1 is particularly responsible for miR-34a up-regulation in PASMCs.

### Inhibition of HDAC1 reduces the ratio of MMP-9/TIMP-1 and MMP-2/TIMP-2 and the expression of collagen I through restoring miR-34a level in MCT-induced PAH rats

**Fig. 5:**
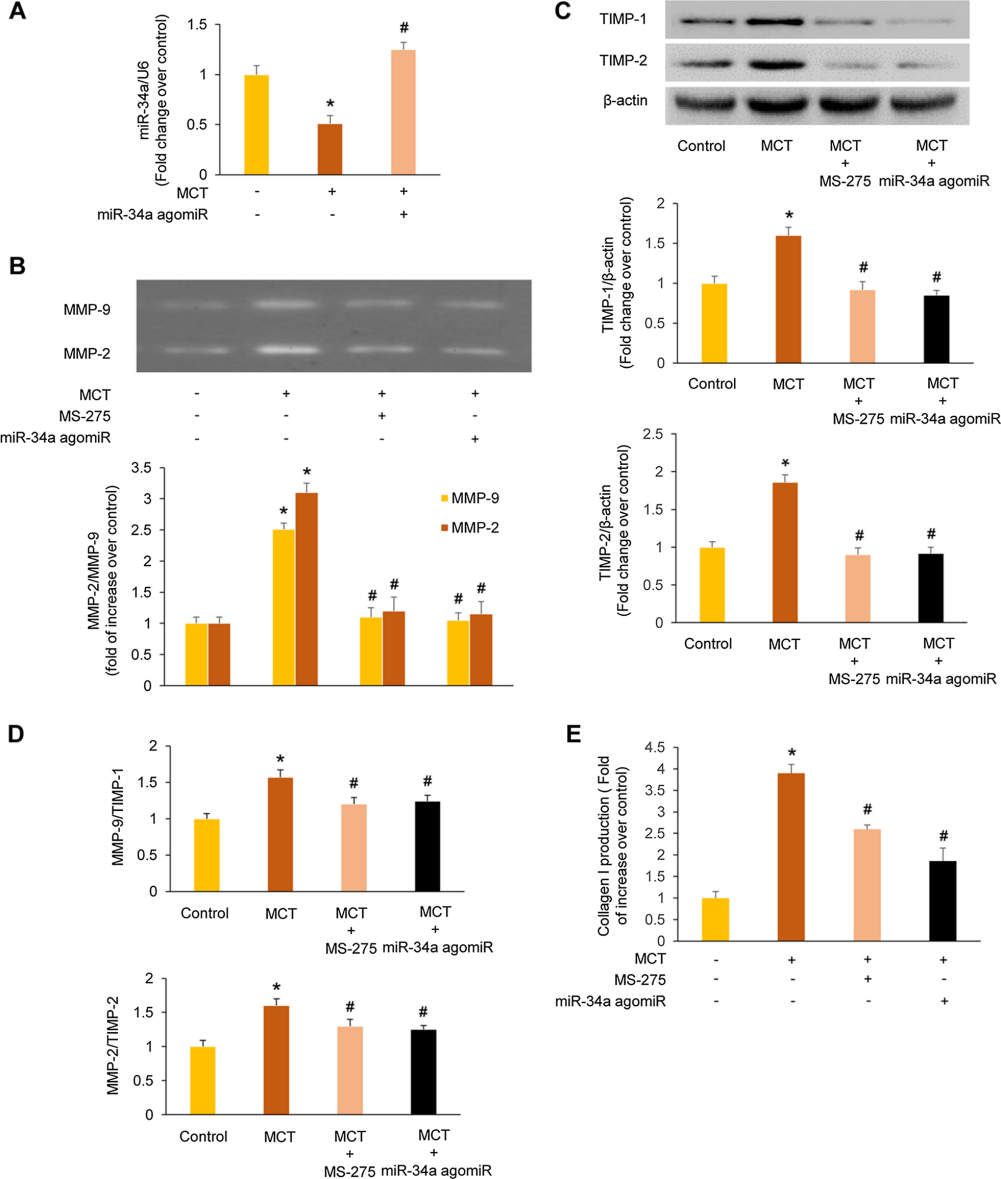
Inhibition of histone deacetylases 1 (HDAC1) reduces the ratio of matrix metalloproteinase 9 (MMP-9)/tissue inhibitor of metalloproteinase 1 (TIMP-1) and MMP-2/TIMP-2 and the expression of collagen I through restoring miR-34a level in monocrotaline (MCT)-induced pulmonary arterial hypertension (PAH) rats. **a** miR-34a level in lung tissues from different groups was determined using quantitative real-time polymerase chain reaction (qRT-PCR) (n = 3). **b** MMP-2/9 activity in lung tissues from different groups was measured using gelatin zymography (n = 3).** c** Protein level of TIMP-1 and TIMP-2 in lung tissues from different groups was examined using immunoblotting (n = 3). **d** Ratio of MMP-9/TIMP-1 and MMP2/TIMP-2 was analyzed in lung tissues from different groups (n = 3). **e** Collagen I production in lung tissues from different groups was determined using enzyme-linked immunosorbent assay (ELISA) (n = 3). *P < 0.05 versus control group; ^#^*P* < 0.05 versus MCT group

To further investigate the mechanism of HDAC1 mediation of MCT-induced collagen deposition, the activity of MMP-2/9 and the expression of TIMP-1/2 were examined in the lung tissues of rats. As shown in Fig. 5A, miR-34a level was down-regulated in MCT-induced PAH model, which was 0.51-fold over control (*P* < 0.05); while treatment of MCT-induced PAH rats with miR-34a agomiR up-regulated miR-34a expression, which was 1.18-fold over control (*P* < 0.05 versus MCT group). Figure 5B indicated that MCT induced a 3.10-fold increase over control in MMP-2 activity (*P* < 0.05) and a 2.51-fold increase over control in MMP-9 activity (*P* < 0.05). MS-275 treatment decreased MMP-2/9 activity in MCT-treated PAH rats to 1.20-/1.10-fold over control (*P* < 0.05 versus MCT-treated group), and administration of miR-34a agomiR reduced MMP-2/9 activity in MCT-treated rats to 1.15-/1.04-fold over control (*P* < 0.05 versus MCT group). Figure 5C showed that the protein levels of TIMP-1 and TIMP-2 increased to 1.60-fold and 1.85-fold over control in the MCT-treated rats (*P* < 0.05), respectively; whereas treatment of MCT-induced PAH rats with MS-275 decreased TIMP-1/2 protein levels to 0.92-/0.90-fold over control (*P* < 0.05 versus MCT-treated group), and administration of miR-34a agomiR reduced TIMP-1/2 protein levels in MCT-treated rats to 0.85-/0.92-fold over control (*P* < 0.05 versus MCT group). Finally, the ratio of MMP-9/TIMP-1 and MMP-2/TIMP-2 was analyzed. As presented in Fig. 5D, the ratio of MMP-9/TIMP-1 and MMP-2/TIMP-2 was 1.57-/1.60-fold over control in MCT-treated rats (*P* < 0.05); MS-275 treatment decreased the ratio of MMP-9/TIMP-1 and MMP-2/TIMP-2 in MCT-treated PAH rats to 1.20-/1.30-fold over control (*P* < 0.05 versus MCT-treated group), and administration of miR-34a agomiR reduced the ratio of MMP-9/TIMP-1 and MMP-2/TIMP-2 in MCT-treated rats to 1.22-/1.25-fold over control (*P* < 0.05 versus MCT group).

Then the expression level of collagen I in the lung tissues was examined by ELISA. As depicted in Fig. 5E, MCT group showed a 3.90-fold increase of collagen I compared with control group (*P* < 0.05). However, treatment of MCT-induced PAH rats with MS-275 and miR-34a agomiR markedly decreased the collagen I level to 2.66-fold and 1.86-fold compared with control group, respectively (both *P* < 0.05 versus MCT group). These results suggest that inhibition of HDAC1 reduces the ratio of MMP-9/TIMP-1 and MMP-2/TIMP-2 and the expression of collagen I through restoring miR-34a level in MCT-induced PAH rats.

### Inhibition of HDAC1 suppresses pulmonary arterial remodeling, excessive ECM accumulation and the increase of RVSP and RVHI through restoring miR-34a level in MCT-induced PAH rats

**Fig. 6:**
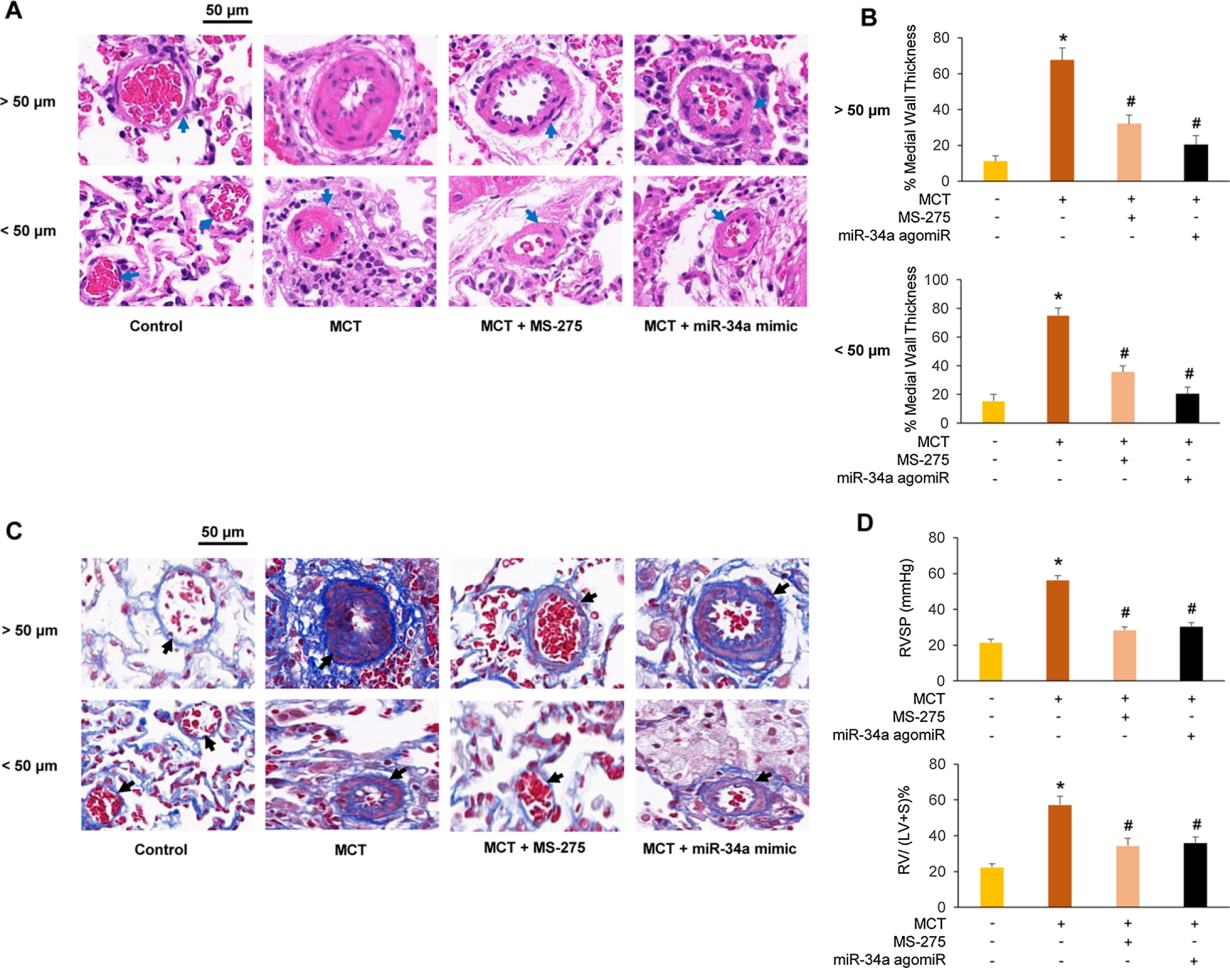
MS-275 suppresses pulmonary arterial remodeling, excessive extracellular matrix (ECM) accumulation and the increase of right ventricular systolic pressure (RVSP) and right ventricular hypertrophy index (RVHI) through restoring miR-34a level in monocrotaline (MCT)-induced pulmonary arterial hypertension (PAH) rats. **a** Hematoxylin and eosin staining of pulmonary arteries (×400 magnification). **b** Quantitative morphometric analysis of the medial wall thickness of small pulmonary arteries. **c** Masson’s trichrome staining of pulmonary arteries (×400 magnification). **d** Changes of RVSP and RVHI in each group, RVHI = RV/(LV + S) (n = 6–8). *P < 0.05 versus control group; ^#^*P* < 0.05 versus MCT group

Pulmonary arterial remodeling plays an extremely critical role in the pathological progress of PAH. The HE staining results indicated that no matter pulmonary vessels with diameters greater than 50 µm or less than 50 µm, both MS-275 and miR-34a agomiR markedly reversed lung vascular remodeling induced by MCT with a reduction of wall thickness in arteries, as presented in Fig. 6A. Quantitative morphometric analysis showed that the percentage of medial wall thickness (%MT) for lung vascular with diameters greater than 50 µm was increased from 11.34 ± 3.25% in control rats to 67.62 ± 6.60% in MCT-treated rats (*P* < 0.05), while administration of MS-275 and miR-34a agomiR reduced the %MT to 32.10 ± 4.95% and 20.43 ± 5.02%, respectively (both *P* < 0.05 versus MCT group). In addition, for lung vascular with diameters less than 50 µm, the %MT was elevated from 15.55 ± 4.52% in control rats to 75.26 ± 5.10% in MCT-treated rats (*P* < 0.05), while administration of MS-275 and miR-34a agomiR reduced the %MT to 35.68 ± 4.35% and 21.89 ± 4.62%, respectively (both *P* < 0.05 versus MCT group), as shown in Fig. 6B. Meanwhile, collagen deposition was determined by Masson’s staining to evaluate the accumulation of ECM components in pulmonary arteries. As indicated in Fig. 6C, collagen deposition in pulmonary vessels with diameters no matter greater than 50 µm or less than 50 µm was significantly increased in the lung tissues of MCT-treated rats compared with control rats. However, the presence of MS-275 or miR-34a agomiR reduced the collagen deposition in pulmonary vessels in MCT-treated rats dramatically. In addition, as depicted in Fig. 6D, treatment with miR-34a agomiR after MCT injection dramatically reduced RVSP from 56.45 ± 2.54 mmHg in MCT-treated rats to 30.56 ± 2.14 mmHg (*P* < 0.05 versus MCT group) and reversed the RV/(LV + S) ratio from 0.57 ± 0.05 in MCT-induced PAH rats to 0.36 ± 0.03 (*P* < 0.05 versus MCT group). These results suggest that inhibition of HDAC1 alleviates pulmonary arterial remodeling, excessive ECM accumulation and the increase of RVSP and RVHI through restoring miR-34a level in MCT-induced PAH rats.

## Discussion

**Fig. 7:**
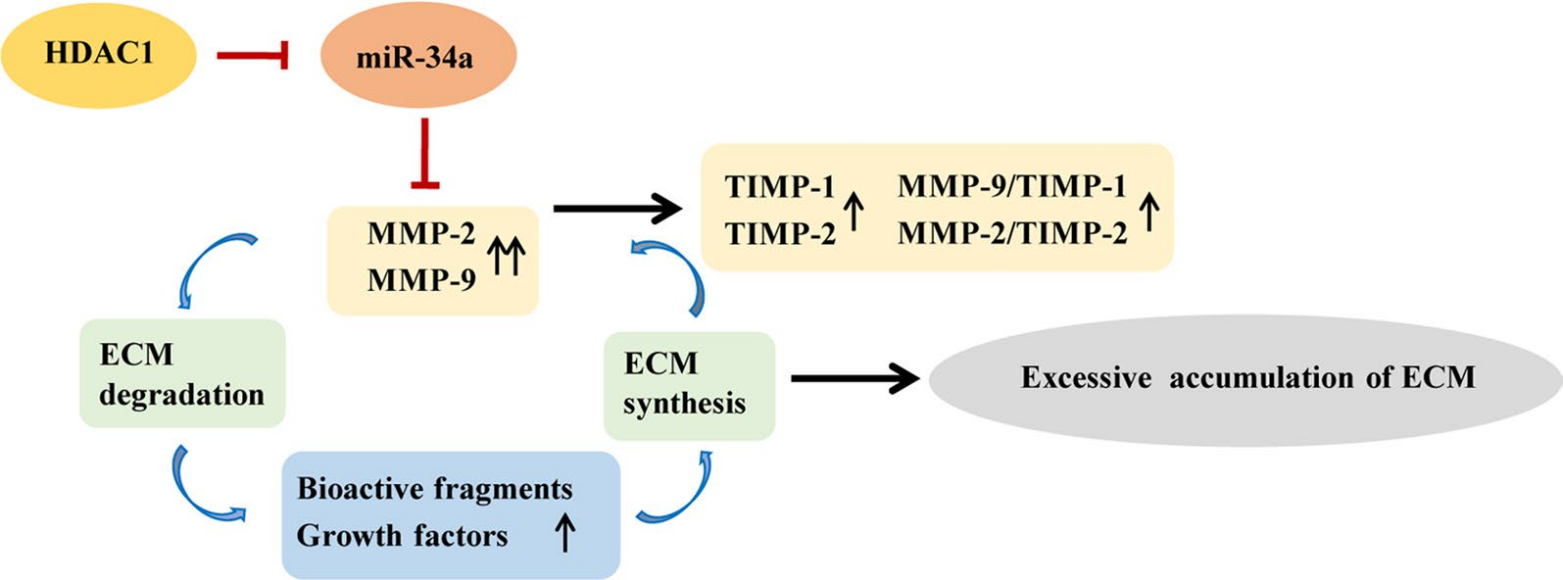
Proposed mechanism for inhibition of histone deacetylases 1 (HDAC1) alleviating monocrotaline (MCT)-induced pulmonary arterial hypertension (PAH). Injection of MCT up-regulates HDAC1 expression, reduces miR-34a level, and subsequently increases the activity of matrix metalloproteinase 2/9 (MMP-2/9). The elevated activity of MMP-2/9 initiates degradation of extracellular matrix (ECM) and finally leads to excessive ECM accumulation through a vicious circle. In addition, tissue inhibitor of metalloproteinase 1/2 (TIMP-1/2) expression is also up-regulated to inhibit excessive MMP activity through a balancing mechanism. However, since the balancing effect is limited, the ratio of MMP-9/TIMP-1 and MMP-2/TIMP-2 is elevated. Inhibition of HDAC1 reverses the process in MCT-induced PAH through restoring miR-34a level

In the present study, we have demonstrated that HDAC1 expression is up-regulated in the lung tissues of MCT-induced PAH rat models, which is coupled to reduction of miR-34a level and elevation of MMP-9/TIMP-1 and MMP-2/TIMP-2 and subsequent increase of collagen I protein. In addition, inhibition of HDAC1 by MS-275 and over-expression of miR-34a by miR-34a agomiR effectively ameliorate excessive collagen I production and pulmonary arterial remodeling, preventing the development of MCT-induced PAH, as shown in Fig. 7. Our results suggest that inhibition of HDAC1 modulates of excessive ECM accumulation and pulmonary vascular remodeling through up-regulation of miR-34a, being of potential value in the management of PAH.

HDAC1 is recruited by multiple transcriptional regulators to a specific genomic region to mediate the repression of corresponding target genes via deacetylation of substrates [21]. Studies have found that HDAC1 plays a vital role in cell proliferation and differentiation, cell cycle regulation as well as tissue transformation and development [22–26]. In addition, HDAC1 is found increased in various cancers, nervous system and heart diseases, and so on [27–29]. HDAC1 level is also elevated in lungs from human idiopathic PAH and rats with PAH; class I HDACs participate in the ECM remodeling of various tissues through different molecular mechanisms [8, 9, 30]. In this study, we used a selective class I HDAC inhibitor MS-275 to examine the effect of HDAC1 on ECM accumulation in pulmonary arteries. Our results demonstrate that inhibition of HDAC1 ameliorates MCT-induced rat excessive ECM accumulation and pulmonary vascular remodeling, indicating that HDAC1 might play an indispensable role in PAH development.

miR-34a causes degradation or translational repression of the target gene through binding to the 3′ untranslated region of mRNAs [31]. miR-34a has been found to participate in various important biological processes, such as cell apoptosis, differentiation and proliferation, as well as tumor suppression, development and metabolism [32–35]. Recent studies have also found that miR-34a level is decreased in the lung tissue of patients and various animal models with PAH; overexpression of miR-34a reverses hypoxia or MCT-induced pulmonary vascular remodeling and PAH [17, 18], suggesting the important role of miR-34a in PAH development. Studies have further shown that HDAC1 inhibits the expression of miR-34a in two ways: HDAC1 binds to the promoter region of miR-34a to directly inhibit gene transcription through deacetylation [15]; HDAC1 directly acts on p53, one upstream gene of miR-34a, and subsequently suppresses miR-34a level [16]. Our present study indicated that both inhibition of HDAC1 activity with MS-275 in MCT-induced PAH rat and loss of HDAC1 with siRNA in PASMCs up-regulated expression level of miR-34a. These results suggest that miR-34a might be a direct target of HDAC1 in pulmonary vascular remodeling, which needs further demonstration in our future studies.

MMP-2 and MMP-9 belong to ECM proteolytic enzymes, which are responsible for collagen degradation, particularly for collagen I degradation [36]. TIMP-1 and TIMP-2 are the small molecular glycoproteins which inhibit the activity of MMP-2 and MMP-9 [37]. Studies have found that elevated MMP activity and consequent ECM remodeling in pulmonary vasculature are involved in the progression of PAH in both experimental models and patients [38–40]. The decreased expression of miR-34a promotes the increased activity of MMP-2 and MMP-9 [41, 42], which is consistent with our present study. Injection of MCT causes response of inflammatory cells and injury of endothelial cells in lungs, which secrete and activate more MMP-2 and MMP-9. MMP-2 and MMP-9 initiate degradation of ECM and subsequent release of bioactive ECM fragments and growth factors, which in turn synthesize and secrete more ECM components. The dysregulated ECM metabolism finally leads to abnormal ECM remodeling through a vicious circle. During this time, the expression of TIMP-1 and TIMP-2 are also up-regulated to inhibit excessive MMP activity through a balancing mechanism [39]. However, since the balancing effect is limited, the ratio of MMP-9/TIMP-1 and MMP-2/TIMP-2 is elevated, which contributes to excessive ECM accumulation and the development of PAH [43].

## Conclusion

The present study demonstrated that inhibition of HDAC1 effectively ameliorates excessive ECM accumulation and pulmonary vascular remodeling in MCT-induced rat PAH, suggesting that inhibiting HDAC1 might be a novel therapeutic strategy in the prevention and treatment of PAH. However, the safety and effectiveness of HDAC1 inhibitors still need to be tested and verified in the further studies.

## Data Availability

All data generated or analyzed during this study are included in this article.

## Abbreviations

%MT: Percentage of medial wall thickness
ECM: Extracellular matrix
ELISA: Enzyme-linked immunosorbent assay
HATs: Histone acetyltransferases
HDAC: Histone deacetylases
HE: Hematoxylin and eosin
LV + S: Left ventricle plus interventricular septum
MCT: Monocrotaline
MMP-2/9: Matrix metalloproteinase 2/9
PAH: Pulmonary arterial hypertension
PASMCs: Pulmonary arterial smooth muscle cells
qRT-PCR: Quantitative real-time polymerase chain reaction
RV: Right ventricle
RVHI: Right ventricle hypertrophy index
RVSP: Right ventricular systolic pressure
TIMP-1/2: Tissue inhibitor of metalloproteinase 1/2
